# Concomitant Guillain–Barré Syndrome in a young Sri Lankan male with severe ulcerative colitis

**DOI:** 10.1186/s12876-022-02455-y

**Published:** 2022-09-05

**Authors:** Jayasundara Mudiyanselage Hishali Dahami Jayasundara, Vajira Tharanga Samarawickrama, Ranjith Peiris, Tilan Aponso, Danushi Abeynayake

**Affiliations:** National Hospital of Sri Lanka, Colombo, Sri Lanka

**Keywords:** Ulcerative colitis, Guillain–Barré Syndrome, Acute Motor Axonal Neuropathy, Inflammatory bowel disease, Case report

## Abstract

**Background:**

Guillain–Barré Syndrome is an immune mediated polyneuropathy. Ulcerative Colitis is an immune mediated chronic inflammatory condition mainly of the large intestine. Guillain–Barré Syndrome can present as a rare extraintestinal manifestation of Ulcerative Colitis when in remission or in a relapse. However, the concomitant presentation of Guillain–Barré Syndrome during a relapse of Ulcerative Colitis is very rare and only a few cases are reported to date.

**Case presentation:**

A 24 year old young male diagnosed of Ulcerative Colitis presented with bloody diarrhea of frequency more than six times a day. He had been in clinical remission even after defaulting treatment for more than a year. He had also noted difficulty in walking prior to admission to the hospital. He was managed as for a severe relapse of Ulcerative Colitis and Guillain–Barré Syndrome. Appropriate management of both the illnesses helped him to recover.

**Conclusion:**

Immune mediated diseases can have rare coexisting presentations. We report a case of Ulcerative Colitis with concomitant Guillain–Barré Syndrome. It is essential to be open minded and timely, appropriate treatment led to successful management of both the illnesses.

## Background

Ulcerative colitis is a chronic inflammatory disease of the colon with relapses and remissions. Its incidence and prevalence are increasing globally including Asia [1]. Neurologic complications of inflammatory bowel disease are not very common [2]. Guillain–Barré Syndrome is considered as one of the rare extraintestinal manifestations of inflammatory bowel disease [3]. Guillain–Barré Syndrome is an immune mediated, monophasic acute paralyzing illness usually provoked by a preceding infection. Out of the different variants of Guillain–Barré Syndrome, Acute Motor Axonal Neuropathy (AMAN) is a primary axonal form of Guillain–Barré Syndrome. It is thought that genetic susceptibility, aberrant self-recognition and immunopathogenic autoantibodies against organ-specific cellular antigens shared by the colon and extra-colonic organs play a role in contributing to the pathogenesis and development of the extra intestinal manifestations [4].The first documentation is in 1985 by Zimmerman where Guillain–Barré Syndrome had occurred in patients who were in remission [5]. To date, only about 9 cases are reported according to the best of our knowledge. We present a young patient who had not received any TNF alpha therapy previously presenting with Acute Motor Axonal Neuropathy (AMAN) variant of Guillain–Barré Syndrome along with a relapse of Ulcerative Colitis, a combination which has not been reported in literature.

## Case presentation

Our patient is a young, 24 year old male, diagnosed with extensive colitis of Ulcerative Colitis in mid- 2020. He had defaulted treatment and not attended to the routine clinic after three months following the diagnosis. However, he had been in clinical remission for almost a year. He was initially treated with oral prednisolone, sulfasalazine and azathioprine. This time he presented with clinical features suggestive of a relapse of severe ulcerative colitis. There was no evidence of toxic megacolon or other detrimental complications. Cessation of smoking was identified as a potential precipitating factor in addition to the poor compliance. He did not have any concomitant extra intestinal manifestations. There had been a marked unintentional weight loss of about 31% from his baseline body weight within a month. One week prior to admission he had noted difficulty in walking but was able to mobilize with support.

He had marked bilateral lower limb edema with general unwellness. He was afebrile and did not have tachycardia, hypotension or a tender abdomen. Lower limb examination revealed bilateral symmetrical proximal more than distal weakness with diminished reflexes and preserved sensation without sphincter involvement. There was no significant muscle wasting or fasciculations. Also, he had symmetrical proximal more than distal upper limb weakness with diminished reflexes and all sensory modalities were intact. His cranial nerves, higher functions and cerebellar examinations were normal. He had a weak neck muscle power and cough effort but was not in respiratory distress. He maintained his vital parameters with no desaturation or fluctuation of blood pressure or pulse rate.

**Fig. 1:**
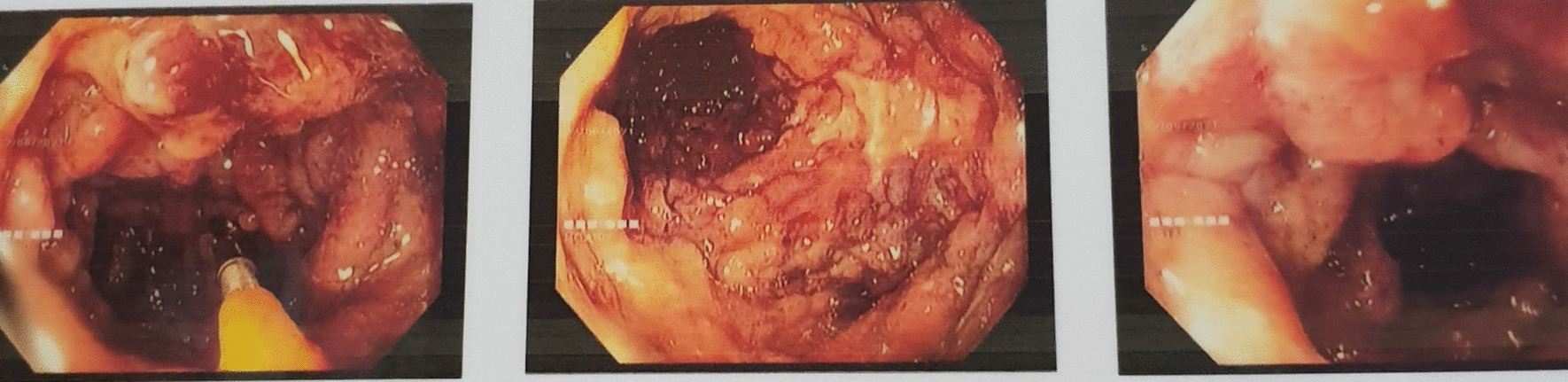
Endoscopic appearance of severe ulcerative colitis

Full blood count revealed a moderate hypochromic microcytic anemia of 9.9 g/dl (reference value 12–15 g/dl). His inflammatory markers such as CRP was 115 mg/L (reference value 0–5 mg/L) and ESR was 45 mm/hr (reference value 1–13 mm/hr).The albumin level was very low of 1.3 g/dl (reference value 3.5–5.5 g/dl). His renal functions were normal and urine full report did not reveal albuminuria. He had a mild hypokalemia and thyroid functions were normal. Fecal calprotectin was positive. Stool cultures excluded other enteric infections and Clostridium difficile toxins were not detected. Flexible sigmoidoscopy revealed severe mucosal inflammation (Mayo score of 3 on endoscopic appearance as shown in Fig. 1) and histology excluded concomitant CMV colitis and confirmed Ulcerative Colitis flare. Nerve conduction study as in Table 1 revealed Acute Motor Axonal Neuropathy type of Guillain–Barré Syndrome. CSF analysis confirmed protein cell dissociation with absent cells and protein of 62 mg/dl. Covid 19 infection was safely excluded as well as other viral aetiologies as CMV, EBV, HIV.

**Table 1:** Nerve conduction study

Site	Latency (ms)	Amplitude (mV)	Area (Vms)	Segment	Distance (mm)	Interval (ms)	NCV (m/s)	NCV N D
**Motor Nerve Conduction Study**								
Ulnar R								
Wrist	2.37	2.86	25.54	Wrist		2.37		
Elbow	6.69	2.82	23.01	Wrist-Elbow	270	4.32	62.5	
Ulnar L								
Wrist	2.49	2.42	12.55	Wrist		2.49		
Elbow	6.81	2.31	12.41	Wrist-Elbow	270	4.32	62.5	
Peroneal R								
Ankle	2.85	900.00 uV	6.78	Ankle		2.85		
Head of Fibula	10.25	880.00 uV	7.92	Ankle-Head of fibula	380	7.40	51.4	
Tibial R								
Ankle	6.95	2.92	11.65	Ankle		6.95		
Peroneal L								
Ankle	2.6	820.00	5.44	Ankle		2.6		
Head of Fibula	10.05	780.00	5.33	Ankle-Head of fibula	380	7.45	51	
Tibial L								
Ankle	4.25	4.56	21.58	Ankle		4.25		

Nerve	Stim.site	F-Lat	F-Lat N.D	M Lat	F-M Lat	F Occurr	
**F Wave Study**							
Tibial R	Ankle	47 ms		5.2 ms			Absent
Tibial L	Ankle	5.2 ms		5.2 ms			Absent
Ulnar R	Wrist	2.6 ms		2.6 ms		0/9.0%	Repeaters
Ulnar L	Wrist	2.75 ms		2.7 ms		0/8.0%	

Site	Latency (ms)	Amplitude	Area	Segment	Distance (mm)	Interval (ms)	NCV (m/s)	NCV N D
**Sensory Nerve Conduction Study**								
Ulnar, R								
Wrist	1.56 ms	26.30 uV	1.33 uVms	Wrist		1.56 ms		
Ulnar, L								
Wrist	1.79 ms	31.10 uV	0.68 uVms	Wrist		1.79 ms		
Sural, R								
Sural	2.42 ms	7.40 uV	0.10 uVms	Sural		2.42 ms		
Sural, L								
Sural	2.21 ms	9.40 uV	0.56 uVms	Sural		2.21 ms		
Radial, R								
Forearm	1.15 ms	33.40 uV	0.75 uVms	Forearm		1.15 ms

**Table 2:** Comparison of investigatios before & after treatment of severe UC

	Pre Treatment	Post Treatment
WBC (× 10^9^/L)	24	13
Hb (g/dL)	9.9	10.9
Plt (× 10^9^/L)	571	437
CRP (mg/L)	115	43
ESR (mm/hr)	45	22
Na (mmol/L)	127	134
K (mmol/L)	3.1	3.8
Albumin (g/dL)	1.3	3.6
Globulin (g/dL)	3.2	3
ALP (mg/dL)	91	98
SGPT (U/L)	34	33
SGOT (U/L)	22	22
GGT	39	36
Cr (mg/dL)	0.8	0.7

The relapse of Ulcerative Colitis was managed with intravenous hydrocortisone of 100 mg every 6 hourly, subcutaneous enoxaparin as for DVT prophylaxis, intravenous fluids and albumin. He showed a significant clinical improvement by day 3 along with a rapid decline of CRP being less than 45. Subsequently, he was managed with oral prednisolone and later on with sulfasalazine 1 g bd and azathioprine 50 mg daily. Since he had many poor prognostic factors such as young age of onset, extensive colitis requiring hospitalization, low albumin, high CRP, Tofacitinib 10 mg twice a day was commenced as he was not a suitable candidate for TNF alpha blockers due to concomitant GBS and other biological agents as vedolizumab or ustekinumab were not available.

Guillain–Barré Syndrome was managed with IV immunoglobulin 0.4 g/kg for five days by which his lower limb proximal muscle weakness improved. His vital capacity and other vital parameters were monitored daily. He received regular chest and limb physiotherapy. By day five there was an improvement in the proximal lower limb weakness.His hematological and biochemical test results too improved as depicted in the following Table 2. He was followed up at the clinic and Ulcerative Colitis was in remission with Tofacitinib, prednisolone tapering regime, sulfasalazine and azathioprine.

## Discussion and conclusions

We present a young male who presented with a relapse of Ulcerative Colitis subsequently developing Guillain–Barré Syndrome. Also he had a significant weight loss and generalized body weakness. Timely, appropriate diagnosis and management aided in marked improvement and recovery. This case demonstrates a rare clinical presentation of coexistent Ulcerative Colitis with AMAN variant of Guillain–Barré Syndrome.

Extra intestinal manifestations occur in 5% to 50% of all patients with inflammatory bowel disease. The severity and occurrence of extra intestinal manifestations and their correlation with intestinal-inflammatory bowel disease activity vary, but most extra intestinal manifestations are directly associated with an ongoing intestinal flare [6].

The association of Ulcerative Colitis with neurologic involvement is rare and often controversial [7]. According to A. Lossos out of the IBD patients who developed neurologic manifestations; 74% had developed after a mean of 5.7 years following development of IBD and only 10% during an IBD exacerbation. The neurologic manifestations documented were in the form of myelopathy, myopathy,myasthenia gravis and cerebrovascular disorders[8]. Peripheral neuropathies related to IBD seems to be more frequent in Ulcerative Colitis, with a reported incidence of 1.9% but it seems that it is associated with a lower rate of demyelinating forms as compared to Crohn’s Disease [9].

Guillain–Barré Syndrome is one form of neurological manifestations which can occur both in remission or a relapse of Ulcerative Colitis[10]. The exact pathogenesis of Ulcerative Colitis with Guillain–Barré Syndrome is unclear.It may be related to the following factors: Ulcerative Colitis-associated vasculitis, post infection immunity, malnutrition, toxic metabolites, vitamin deficiency, and thrombotic disease[11]. It is also postulated that since both Ulcerative Colitis and Guillain–Barré Syndrome are autoimmune diseases there may be similar autoimmune mechanisms in the development of both these diseases. However,association of Guillain–Barré Syndrome and Ulcerative Colitis is extremely rare and only a few cases have been reported [12, 13]. There are different variants of Guillain–Barré Syndrome of which Acute Motor Axonal Neuropathy (AMAN) is one such type. Our case was an Acute Motor Axonal Neuropathy (AMAN) form of Guillain-Barré Syndrome with a relapse of Ulcerative Colitis which has not been reported up to date so far. Most cases have antecedent infection with *Campylobacter jejuni* and many have antibodies directed towards GM1 ganglioside-like epitopes.The mechanism of nerve-fiber injury has not been defined yet. Acute Motor Axonal Neuropathy (AMAN) is a novel disorder caused by an antibody- and complement-mediated attack on the axolemma of motor fibers [14]. The nerve conduction study was in favor of axonal injury in our patient.

Infliximab, a Tumor Necrosis Factor (TNF) alpha blocker, is known to be an effective treatment for Ulcerative Colitis. There are many cases documented in the literature mainly by the US Food and Drug Administration where Guillain–Barré Syndrome had developed after the initiation of anti- Tumor Necrosis Factor (TNF) alpha therapy [15, 16]. Interestingly, our patient had not received infliximab therapy at any time.

Vedolizumab,is an anti integrin, a humanized IgG1 monoclonal antibody against a4b7 integrin that inhibits leukocyte adhesion of MAdCAM-1 specific to the bowel. It is recommended for the treatment of moderate to severe ulcerative colitis for those who are not responding to one or more conventional treatment such as steroids, immunosuppressive agents, or TNF blockers [17]. One of its rarest adverse reaction is progressive multifocal leukoencephalopathy, which a demyelinating condition of the central nervous system. Ustekinumab is another monoclonal antibody used in treating moderate to severe ulcerative colitis for those who have failed to recover from conventional and /or biological therapies.It acts by blocking the p40 subunit of IL-12 and IL-23 and can be used in naïve subjects or patients previously exposed to biologics [18].The adverse effects of ustekinumab on the nervous system are very minor. However, both these agents are scarce and very costly in a developing country as ours. Our next choice was Tofacitinib which is an orally administered small molecule and a Janus kinase inhibitor. Tofacitinib is one of the drugs emerging into the limelight for the management of moderate to severe Ulcerative Colitis. It is known to be cost effective and also effective in achieving endoscopic response, endoscopic remission, and mucosal healing [19].

It is important to exclude other causes of weakness in a patient presenting with diarrhea; mainly electrolyte imbalances, endocrine disorders as hypothyroidism, thyrotoxicosis and iatrogenic Cushing’s syndrome. Finally, the clinical picture and the relevant investigations directed us for appropriate and timely management of our patient. Thus, our case highlights the importance of thorough clinical examination and being keen on the rare manifestations of common illnesses.

## Data Availability

Not applicable.

## Abbreviations

DVT: Deep vein thrombosis
CSF: Cerebro spinal fluid
IBD: Inflammatory bowel disease
CRP: C reactive protein
